# Evolution of patients' complaints in a French university hospital: is there a contribution of a law regarding patients' rights?

**DOI:** 10.1186/1472-6963-9-141

**Published:** 2009-08-06

**Authors:** Camila Giugliani, Nathalie Gault, Valia Fares, Jérémie Jegu, Sergio Eleni dit Trolli, Julie Biga, Gwenaelle Vidal-Trecan

**Affiliations:** 1 Public Health Department, School of Medicine, Paris Descartes University, Assistance Publique – Hôpitaux de Paris, Cochin – St. Vincent de Paul Hospital Group, Paris, France; 2 Post-Graduate Program of Epidemiology, Social Medicine Department, School of Medicine, Federal University of Rio Grande do Sul, Porto Alegre, Brazil; 3 Department of Patients Rights, Assistance Publique – Hôpitaux de Paris, Cochin – St. Vincent de Paul Hospital Group, Paris, France

## Abstract

**Background:**

Legislative measures have been identified as one effective way of changing attitude or behaviour towards health care. The aim of this study was to describe trends in patients' complaints for medical issues; to evaluate the contribution of a law regarding patients' rights, and to identify factors associated to patients' perception of a medical error.

**Methods:**

Patients with a complaint letter for medical issues in a French university hospital were included. Trends in complaint rates were analysed. Comparisons were made between a first (1998–2000) and a second (2001–2004) time period, before and after the diffusion of the law, and according to the perception of a medical error.

**Results:**

Complaints for medical issues increased from 1998 to 2004. Of 164 complaints analysed, 66% were motivated by the perception of a medical error (47% during the first time period vs. 73% during the second time period; p = 0.001). Error or delay in diagnosis/treatment and surgical/medical complication were the main reasons for complaints. Surgical departments had the higher number of complaints. Second time period, substandard care, disability, and adverse effect of a health product were independently associated with the perception of a medical error, positively for the formers, and negatively for the latter.

**Conclusion:**

This study revealed an increase with time in the number of complaints for medical issues in a university hospital, as well as an increase in the perception of a medical error after the passing of a law regarding patients' rights in France.

## Background

Patient safety and risks of inpatient care are current issues of concern worldwide and should be a declared priority within health care organizations, since adverse events account for unacceptable high levels of patient morbidity and mortality [1-3], varying from 3,7% in France or in the New-York State [4-6] to around 10% in Great-Britain or New-Zealand [5-8]. Many countries have guaranteed patients' right to process for resolving dissatisfactions with health care providers. The United States, for example, since November 1997 have included an aspirational statement in the Consumer Bill of Rights regarding this issue [9]. In this country, where iatrogenesis is the third leading cause of death [10], increasing patients' participation in their care, reducing health care errors, and ensuring the appropriate use of health care services are among the national aims for improving the quality of health care [11,12]. Other countries, such as Sweden, the United Kingdom, Italy, Spain, and New Zealand also have a specific legislation regarding patient's protection and safety [13,14]. In France, considerable efforts have been made in the past 20 years to improve in-hospital safety management through laws and regulations [15-17]. Furthermore, patients found their rights supported by a law [17] developed in 2001 by the health authorities and voted in by Parliament on 4 March 2002. This law concerned among others: the respect of dignity and the absence of discrimination regarding patients' care, better identification and management of the consequences associated with poor sanitation (right for compensation for no-fault medical accidents and better access to medical insurance for persons with a serious health risk), and assurance of direct access to medical records. Moreover, a national institution depending on the Ministry of Health was created (National Organism of Financial Compensation of Medical Accidents, iatrogenesis and nosocomial infections, the ONIAM [Office National d'Indemnisation des Accidents Médicaux, des affections iatrogènes et des infections nosocomiales]. This institution is in charge of the organisation of the plan for out-of-court resolution of medical accidents and of financial compensation of patients in case of no-fault medical accident. In addition, the creation of a commission of relations with patients became a requirement to every hospital. All these measures may have contributed to an increased expectation of patients towards the health system and may have facilitated the act of officially addressing a complaint.

A complaint can express grief or resentment, resulting in some cases in financial compensation. However, some studies have found that the main reason for patients to file a complaint was not the desire for financial compensation but to expose that things went wrong, and to get an answer from the hospital or doctors stating that the reoccurrence of the situation will be prevented [18,19].

Legislative measures have been identified as one effective way of perpetuating a change in attitude or behaviour regarding health care [20]. In terms of patients' rights within the health system, we hypothesized that the law has facilitated the complaining process. However, published data regarding the effects of legislative measures on patients' complaints is limited. Thus, this study reports an analysis of patients' complaints regarding medical issues, from the patient's perspective, in a university hospital during a period of 6.3 years (1998 through 2004), including (1) a description of their trends, (2) an identification of factors associated with patients' perception of a medical error, and (3) an evaluation of the contribution of the law voted on March, 4^th ^2002.

## Methods

### Study design

We conducted an analysis of patients' complaint letters received by the Direction of a university hospital from 01/01/1998 to 30/03/2004, considering the patient's perspective.

### Setting

The study was performed in a hospital belonging to the Assistance Publique – Hôpitaux de Paris, the Public Network of Parisian Hospitals (41 hospitals). This hospital complex (1,316 short-stay beds at two different locations) houses all major medical and surgical departments, except nephrology, neurosurgery, and cardiovascular surgery. In 1999, a paediatric hospital joined the hospital complex. A medical department of public health is in charge of quality and safety of health care. The hospital's Direction includes a Department of Patient's Rights which is responsible for handling all written complaint letters coming from four main sources: the litigation department of the Public Network of Parisian Hospitals; the court, the President's administration of justice, which transmits complaints to the litigation department of the Public Network of Parisian Hospitals; the hospital's clinical departments, especially the Emergency department; and the Department of Patient's Rights itself, which also receives complaints written by all the previous sources.

The department of Patient's Rights investigates every complaint and tries to solve it out of court. In case of failure, the case is transmitted to the litigation department of the Public Network of Parisian Hospitals. In case of no-fault medical accident, patients can refer their case to a regional commission of conciliation, which decides the allocation of a financial compensation without litigation [17].

### Study population

Every inpatient with a written complaint regarding medical issues, with or without legal involvement, coming in the hospital's Department of Patient's Rights within the study period was included. Both inpatients and outpatients having visited the Emergency Department were considered eligible. Complaints exclusively for financial issues (e.g., hospitalisation fees), accommodation, hosting quality, and organisational matters, were excluded from the sample by the staff of the Department of Patient's Rights. Complaints that were not excluded for these criteria were considered as complaints for medical issues.

### Data collection

The number of hospital admissions (excluding day-care) and of visits to the Emergency Department was drawn from the annual hospital activity reports. Within the study period, the hospital registered 705,632 both outpatient visits to the Emergency Department and inpatient admissions in other clinical departments. The Department of Patient's Rights received a total of 2,116 complaint letters, of which 164 were for medical issues, selected by the Director of quality and patients' rights and one of the investigators (VF). We chose to collect only the complaints for medical issues because these are more related to health providers' practice, consequently, medical errors, which, amongst the reasons of complaints, is the main focus of this study. Information contained in the complaint letters for medical issues was collected using an anonymous semi-structured form filled in by the same investigator from the medical department of public health. This form was conceived according to the content analysis of the complaints. Data collected included patient's demographics, type of issue, type of department, patient's perception of a medical error, type of patient's outcome after the perceived medical issue and demand for financial compensation. The medical department of public health was in charge of coding the written complaints, and handled the raw data. Complaints were classified by confronting blind conclusions of three investigators from the medical department of public health (GVT, SET et JB) for three main issues: the perception of a medical error, the reason of complaint and the consequences of the accident leading to the written complaint. In case of disagreement between them, a case discussion took place to reach a consensus opinion.

### Definition of variables

The complaint rates were calculated by dividing, first, the number of complaints for medical issues by the total number of complaints received by the Department of Patient's Rights, and second, the number of complaints for medical issues by the hospital activity.

We defined two different time periods, considering the large amount of media coverage related to the passing of the law in 2001, previously to its implementation: 1998–2000 ("first period") and 2001–2004 ("second period"). Another definition could have been considered (1998–2001 and 2002–2004, for instance); but the "first period" was chosen after a sensitivity analysis of the results for two time periods (i.e., 1998–2000 and 1998–2001). The comparison of complaint's characteristics between 1998–2001 and 2002–2004 lead to non significant differences. The results of our analysis were sensitive to the changes in thresholds of time periods.

Content analysis of the letters allowed us to classify reasons for complaints according to patient's perspective in two different variables:

(1) The types of issue were listed as error or delay in diagnosis or in treatment (i.e. wrong or delayed diagnosis or medication, resulting in adverse outcome), nosocomial infection, the adverse effects of a health product (i.e., blood product, drug, graft, medical device), results of a surgical or a medical complication, information or surveillance problem (i.e. lack of interest, attention or care), non performance of a required procedure or treatment and other. Error or delay in diagnosis or in treatment, and information or surveillance problem were considered as parts of a "substandard care" category (i.e. procedures not performed according to standard of care). Results of a surgical or a medical complication and nosocomial infection were grouped together in a "complication of care" category.

(2) The outcomes were listed as death, disability, surgery (operation or re-operation), prolonged care (e.g. hospital stay), failure to achieve standard of care, and other. Surgery, prolonged care and failure to achieve standard of care were considered as parts of a "prolonged care" category.

We defined two types of departments, those functioning essentially on an emergency basis (adult and paediatric emergency departments, obstetrics, gynaecology, and neonatology), and the others.

### Statistical Analysis

First, trends in complaint rates for medical issues were examined on a yearly basis (number of complaints among hospital activity and complaints for medical issues among all complaints). The association between the rates of complaints and years was measured using Pearson correlation coefficient. We further analysed the evolution of the number of complaints according to time (years) and the existence of the law, using a Poisson regression, however, the Variance Inflation Factor (VIF) and tolerance exploring the co linearity between these variables led us not to use the results of this model.

Second, we performed a descriptive study of complaint letters, using proportions and their 95 percent confidence intervals (95% CI). Third, we compared the characteristics of the written complaint between the first and the second periods, and according to the perception of a medical error, using chi-square tests.

We further explored the association of these differences with the perception of a medical error using a logistic regression model. Variables with *p *< 0.05 were included in the model, run with a conditional backward stepwise procedure. The associations were expressed as odds ratios (ORs) with their 95% CI. Diagnosis of the regression model and robustness were checked.

All tests were two-tailed, and statistical significance was set at *p *< 0.05. The software SPSS^® ^for Windows™, version 12.0 (Copyright© SPSS Inc., 2003) was used for data analysis.

### Ethical considerations

This was a study of patients' complaints, with anonymous data collection, thus demanding no specific ethics approval.

## Results

### Trends

**Figure 1:**
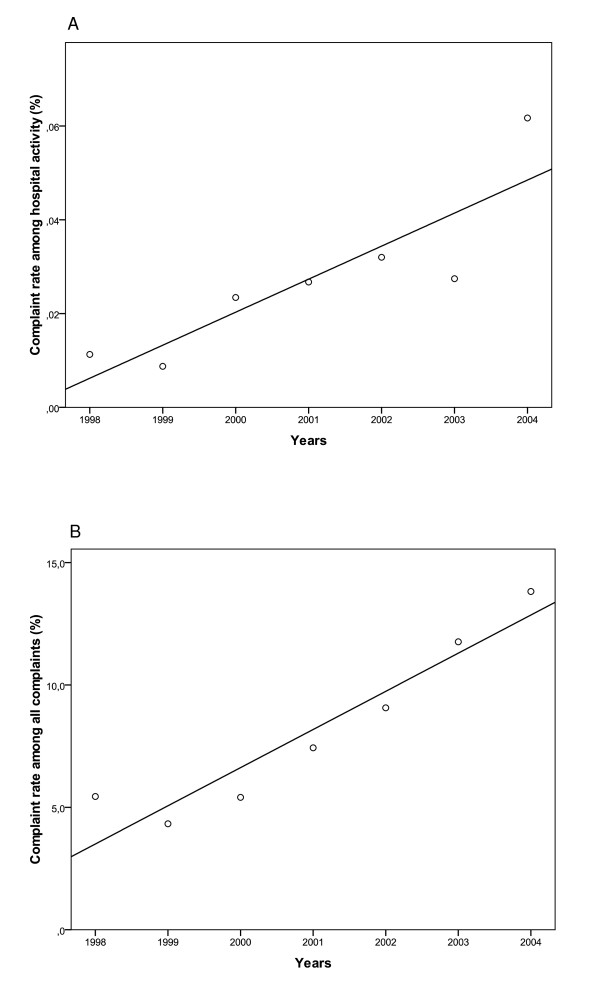
**Evolution of complaints for medical issues among hospital activity (a) and complaints for medical issues among all complaints (b) between 1998 and March 2004**.

The rate of complaints for medical issues according to hospital activity increased yearly (Table 1) with a correlation coefficient of Pearson of 0.87 (*p *= 0.01). The rate of complaints for medical issues among all complaints also increased, from 5.4% in 1998 to 14.2% in 2004 (Table 1), with a coefficient of Pearson of 0.95 (p = 0.001). The evolutions of complaints are illustrated in a clear trend (Figure 1).

**Table 1:** Rates of complaints in a university hospital of the Public Network of Parisian Hospitals from 1998 to 2004

Year	Complaints for medical issues among all complaints n/N (%)	Complaints for medical issues among hospital activity n/N* (%)
1998	11/202 (5.4)	11/97 417 (0.011)
1999	11/254 (4.3)	11/125 756 (0.009)
2000	25/462 (5.4)	25/106 688 (0.023)
2001	31/417 (7.5)	31/115 983 (0.027)
2002	37/408 (9.2)	37/115 618 (0.032)
2003	32/272 (11.9)	32/116 619 (0.027)
2004^†^	17/123 (14.2)	17/27 551 (0.062)

* N = emergency visits and inpatients, excluding one-day hospitalizations

^† ^Only the period of January through March has been studied

### Characteristics of study population and complaints

Women accounted for 63% (95% CI = 55% – 70%) of complaint letters for medical issues. Median age was 39 years (range: 0 – 88). Patients less than 15 accounted for 22% (95% CI = 16% – 30%) of letters. The complaint was written by a lawyer in 42% of cases (95% CI = 35% – 50%), whereas the affected patient was the author in 41% of cases (95% CI = 33% – 49%). Seventeen percent of letters were written by relatives (95% CI = 11% – 23%). Eleven complaints (6.7%, 95% CI = 3.4% – 11.7%) were referred to the regional commission of conciliation. Among them, only one was further referred to a court.

Complaint letters were mostly due to error or delay in diagnosis or treatment (n = 40, 24%, 95% CI = 18% – 32%), results of a surgical or a medical complication (n = 40, 24%, 95% CI = 18% – 32%), or adverse effects of a health product (n = 27, 17%, 95% CI = 11% – 23%). The other issues reported were the non performance of a required procedure or treatment or other (n = 23, 14%, 95% CI = 9% – 20%), information or surveillance problem (n = 18, 11%, 95% CI = 7% – 17%), and nosocomial infection (n = 16, 10%, 95% CI = 6% – 15%). In 108 patients, (66%, 95% CI = 58% – 73%), the complaint was perceived as a medical error. Complaints for medical issues were more frequent in the surgical and obstetrical departments (respectively 39% and 23%), so as the medical error perceived by the patient (respectively 30% and 28%). Death was reported in 26 complaint letters (16%, 95% CI = 11% – 22%). For 45 patients (27%, 95% CI = 21% – 35%), disability was the final outcome. The other reported outcomes were prolonged care (n = 11, 7%, 95% CI = 3% – 12%), surgery (n = 10, 6%, 95% CI = 3% – 11%), failure to achieve standard of care (n = 9, 5%, 95% CI = 3% – 10%) and others or unknown (n = 63, 38%, 95% CI = 31% – 46%).

### Comparisons of the characteristics between the two periods (Table 2)

**Table 2:** Comparison of complaints for medical issues between two time periods (1998–2000 and 2001–2004) in a university hospital of the Public Network of Parisian Hospitals*

	**Time period**		
**Variable**	First n/N (%)	Second n/N (%)	p
Age > 39	25/40 (63)	40/95 (42)	**0.030**
Male gender	19/47 (40)	42/117 (36)	0.590
Departments functioning on an emergency basis^†^	12/47 (26)	52/117 (44)	**0.025**
Perception of a medical error by the patient	22/47 (47)	86/117 (73)	**0.001**
Reason of complaint			0.119
*Error/delay in diagnosis or treatment*	8/42 (17)	32/117 (27)	
*Nosocomial infection*	6/42 (13)	10/117 (9)	
*Adverse effect of a health product*	10/42 (21)	17/117 (15)	
*Result of a surgical/medical procedure*	16/42 (34)	24/117 (21)	
*Information or surveillance problem*	2/42 (4)	16/117 (14)	
*Non performance of a required procedure or treatment, or others*	5/42 (11)	18/117 (15)	
Consequences			0.484
*Death*	9/47 (19)	17/117 (15)	
*Disability*	10/47 (21)	35/117 (30)	
*Prolonged care*	7/47 (15)	23/117 (20)	
*Other or unknown*	21/47 (45)	42/117 (36)	

* N is according to each variable, depending on non-missing values.

†Obstetrics, gynecology, neonatology and Emergency Departments

Complaints regarding younger patients, departments functioning essentially on an emergency basis and perception of a medical error were significantly more frequent in the second period.

### Comparisons of patient's characteristics according to the perception of a medical error (Table 3)

**Table 3:** Characteristics of patients, hospitalization and complaints associated with the perception of a medical error in a university hospital of the Public Network of Parisian Hospitals (conditional backward stepwise logistic regression model)

	**Perception of a medical error**			Crude OR*	Adjusted OR
**Variable**	Yes n/N (%)	No n/N (%)	p	(95% CI^†^)	(95% CI)
Age > 39	39/87 (45)	26/48 (54)	0.299		
Male gender	36/108 (33)	25/56 (45)	0.155	0.6 (0.3 – 1.2)	-
Departments functioning on an emergency basis ^‡^	50/108 (46)	14/56 (25)	**0.008**	**2.6 (1.3 – 5.3)**	-
Financial compensation	15/64 (23)	2/26 (8)	0.136		
Second period (2001–2004)	86/108 (80)	31/56 (55)	**0.001**	**3.2 (1.6 – 6.4)**	**2.4 (1.0 – 5.3)**
Type of issue			**<0.001**		
*Substandard care *^§^	48/108 (44)	10/56 (18)		**2.4 (1.3 – 4.5)**	**2.2 (1.1 – 4.2)**
*Complication of care *^¶^	31/108 (29)	24/56 (43)		0.6 (0.4 – 1.1)	0.6 (0.4 – 1.2)
*The adverse effect of a health product*	10/108 (9)	18/56 (32)		**0.3 (0.1 – 0.5)**	**0.3 (0.2 – 0.7)**
*Non-performance of a required procedure or treatment, other*	19/108 (18)	4/56 (7)		-	-
Outcomes			**0.004**		
*Death*	17/108 (16)	9/56 (16)		0.85 (0.4 – 1.7)	0.7 (0.3 – 1.5)
*Disability*	38/108 (35)	7/56 (13)		**2.4 (1.2 – 4.8)**	**2.4 (1.1 – 4.8)**
*Prolonged care*	21/108 (19)	9/56 (16)		1.0 (0.5 – 2.0)	1.0 (0.5 – 2.1)
*Other, unknown*	32/108 (30)	31/56 (55)		-	-

* Odds Ratios

† 95% Confidence Interval

‡ Obstetrics, gynecology, neonatology and Emergency Departments

§Error/delay in diagnosis or treatment, information or surveillance problem

¶Results of a surgical or a medical complication, nosocomial infection

Complaints regarding departments functioning essentially on an emergency basis, and made in the second period were significantly more often associated with the perception of a medical error. Complaints due to substandard care and complaints where disability was the main outcome were significantly associated with the perception of a medical error, whereas complaints due to the adverse effect of a health product were associated with the non-perception of a medical error. After adjustment, the second period, substandard care and disability as the main outcome were positively associated with the perception of a medical error, whereas complaints due to the adverse effect of a health product were negatively associated.

## Discussion

In this study based on the analysis of written complaints to the Department of Patient's Rights of a French university hospital, we found a time trend in the increments of the rate of complaints for medical issues related to the hospital activity and in the proportion of complaints for medical issues among all complaints. On the one hand, we observed that complaints referring to substandard care or complaints from patients whose outcomes had resulted in disability were positively associated with the perception of a medical error and this change appears to have been associated to the release of a law regarding patients' rights and quality of the health care (implemented in 2002). On the other hand, complaints referring to the adverse effect of a health product were negatively associated with the perception of a medical error. Moreover, complaints for medical errors were more frequent in the second period (2001 – 2004).

To our knowledge, this study is among the first analyses of patients' complaints in a university hospital in France. The examination of patients' complaints has been used by others [21] as patient's safety indicators in health care facilities. In other countries, some studies have concluded that the number of complaints increased [8,22-24]. In our study, an incremental impact of the law on the overall number of complaints could not be detected; the increment with time that we found might be a trend over a long period. In fact, one study, in the United Kingdom [23], found an increase of complaints after the implementation of a new complaint procedure.

Several reasons (e.g., improvement in organisational aspects due to engagement in accreditation procedures, reduction in length of hospital stay) could have led to a decrease in the number of complaints, especially complaints for medical issues. Nevertheless, we found an increasing number of complaints for medical issues despite organizational improvement, which suggests that this kind of complaint may not depend on organizational aspects. Other contributors to the increase in the proportion of complaints for medical issues among all complaints might be the presence of less inappropriate complaints for all matters, and a greater search for guilt within complaints. Other causes should still be considered, such as patients' greater consciousness of their rights and broader public debate about adverse events and health care errors, leading to a more demanding attitude towards the health care system (e.g. demand for financial compensation). The law may have lead the complainants to identify complaints as caused by a medical error perhaps because people think that a complaint for a medical error is more likely to lead to a financial compensation, even if there are other important reasons motivating people to complain, as mentioned above. We should keep in mind that a multivariate regression could have provided more accurate results, in particular, the independent effect of the time and that of the law. However, the small number of complaints each year, and the colinearity between the time variable and the law variable, led to misleading results that cannot be interpreted. In our study, we observed an increase of complaints written by lawyers; the reason could be a better and facilitated access to medical records and the current trend in France to increasing lawsuits for medical errors.

Complainants in this study were mainly middle-aged women. Such characteristics are supported by other publications [18,19,25,26]. Moreover, our findings confirmed the results found by other authors [6,8,27], in that the departments mostly affected by patients' complaints were surgery, obstetrics, gynaecology and neonatology.

We observed that reasons for complaints were mainly a result of a surgical or a medical complication, such as in an Australian study [28], and "substandard care" (error or delay in diagnosis or treatment, and information or surveillance problem). One American study [27] of the causes of adverse events in 1,047 inpatients reported the adverse effects following a particular procedure as one of the most common causes. Other authors have also reported concerns with standard of care among the main reasons for complaints [22,29], reflecting increased awareness of desirable standards of quality of care, or indeed patients' high expectations. Two studies [19,22] reported information problems within the three main reasons for complaint. Regarding the adverse effects of a health product, the results of a national study in French health facilities also reported this as the second most common cause of serious adverse events [6]. We found no other study reporting this particular reason of complaint. One possible explanation is that this could be accounted apart from complaints regarding hospital care directly, belonging, thus, to other statistics on adverse events.

In our study, a significant number of complaints were associated with death or disability (16% and 27% respectively). A recent French national study [6] reported 8% and 22% of death and disability, respectively, among the outcomes of serious adverse events, not far from the figures found in some British and American hospitals [4,8]. The higher rate of death found in our study could be explained by the adopted perspective (patients or relatives); actually, death is an outcome more traumatic than others and, thus, possibly more often the object of a complaint, even if it is not the result of an adverse event.

The impact of complaints can be diverse. Whilst complaints can be intended to promote better quality of health care and safer practices, two studies from New Zealand have shown the negative impacts of patients' complaints on surgical trainees training and performance (e.g. more defensive practice, less enjoyment and more stress) [25], and on surgeons practice (increased defensiveness and not learning from complaints) [26]. These studies have also shown that there is usually no appropriate support or guidance to deal with the harmful effects of complaints on medical practice, and that organisational support is needed, as well as an environment that encourages open disclosure and learning from mistakes.

In our study, the majority (66%) of complaints for medical issues was viewed by the patient as medical errors, whether this perception was founded or not. Such a perspective is not rare. In a study of public opinion in the United States [30], a national telephone survey performed in 1997, 42% of respondents reported that they or their close friends and relatives had already experienced a medical mistake. Moreover, the professional and the patient may have different opinions regarding the incident; what a patient believes to be an error may not have been perceived as such by the professional, or may have been caused by a chain of events [19]; usually this is a very complex matter.

In terms of the time period, we found that the perception of a medical error by the patient was more frequent on the second period of our study, which might reflect a change in the attitude of patients after the implementation of a legislative measure. We observed that patients were younger in the second period (2002–2004), a fact that can be explained by the addition of a paediatric hospital to the medical centre in 1999. However, this difference regarding the patient population had no influence in the perception of a medical error. The association that we observed between complaints for substandard care and perception of a medical error could be explained by an increase in the population's awareness of desirable standards of care.

On the one hand, we noticed that complaining for disability was more often associated with the perception of a medical error. This could be explained by the fact that, in this case, patients are more demanding for a financial compensation, as an amend to a functional loss. On the other hand, death was not associated with the perception of a medical error, probably because in this case, families are more often demanding for explanations and apologies.

Complaints are frequently considered the most acceptable option for expressing frustration and disappointment with the health care provided, but they might also be intended to promote better quality of care, in the sense of minimizing preventable health care related injuries. Moreover, in case of litigation, complaints can lead to economic sanctions on those who provide substandard care that leads to injuries.

Our study has some limitations. Since we found no typology for reason of complaints, we adapted typologies both from our analyses of complaint letters and from other studies [7,8,28]. Moreover, the classification method with three investigators could have led to classification bias. Indeed, our study intended to contain a qualitative analysis of written complaints. Since, in case of disagreement, a consensus opinion between investigators was reached, no interrater agreement was calculated. Other limitations were the small size of the sample, leading to a possible lack of power for detecting factors associated with the observed findings, retrospective design of the study and only one hospital investigated, so that our sample might not be considered representative of French inpatients' complaints. However, according to the data of the regional hospitalisation agency , the offer of care in our hospital may be considered representative of all teaching hospitals in Paris. Our study of complaints only captures an unknown fraction of patients that would complain of a medical error. As previously described [31], one reason is that "the complaint process is emotionally and financially costly, confusing, cumbersome and difficult to access". Since we analyzed only the written complaints arriving to the Department of Patient's Rights, a small number of complaints may not have been captured (e.g., those received by clinical departments and not transferred to the Department of Patient's Rights). We suppose that those non captured complaints were quantitatively and qualitatively minor, since in case of a major issue, patients would prefer that their complaint be managed by a hierarchic authority. Moreover, the staff of the Department of Patient's Rights is in charge of answering the patient's complaint and there may be no interest for this Department in underestimating the number of complaints received. Finally the design of this study did not allow to investigate, the factors influencing patients in actually deciding to file a complaint, neither could it state the causal relationship between the introduction of a new law and the number of complaints; nevertheless, we could clearly notice that the increase is contemporary to the implementation of the law.

## Conclusion

In conclusion, this study reveals an increase in the number of complaints for medical issues in a university hospital in Paris over time, as well as an increase of the perception of a medical error after the diffusion of a law regarding patients' rights that may have contributed to this trend. Thus, we believe that the 2002 law regarding patient's rights may have contributed to an increase in complaints for medical issues due to a change in people's attitude towards the complaining process.

## Competing interests

The authors declare that they have no competing interests.

## Authors' contributions

CG and NG led the data analyses and the writing of the manuscript. VF, JJ and SET were involved in the study initiative and in data collection and preliminary analyses. JB participated actively in the management of databases and data analyses. GVT was the coordinator of the study and supervised all stages. All authors read and approved the final manuscript.

## Authors' information

GVT can be reached via the following email address - gwenaelle.vidal-trecan@parisdescartes.fr

## Pre-publication history

The pre-publication history for this paper can be accessed here:


